# 6′-Amino-3′-methyl-11*H*,2′*H*-spiro­[indeno­[1,2-*b*]quinoxaline-11,4′-pyrano[2,3-*c*]pyrazole]-5′-carbonitrile ethanol monosolvate

**DOI:** 10.1107/S1600536812026992

**Published:** 2012-06-20

**Authors:** Jia Zheng, Bin Li, Yi-Qun Li

**Affiliations:** a Department of Chemistry, Jinan University, Guangzhou 510632, People’s Republic of China

## Abstract

In the title spiro­indeno­quinoxaline compound, C_22_H_14_N_6_O·C_2_H_6_O, the five-membered ring of the indene unit and the pyran ring are perpendicular [89.11 (3)°]. In the crystal, N—H⋯N hydrogen bonds connect the spiro­indeno­quinoxaline mol­ecules, and the ethanol solvent mol­ecules complete the hydrogen-bond network *via* O—H⋯N and N—H⋯O inter­actions.

## Related literature

For general background to spiro compounds and their biological activity, see: Pradhan *et al.* (2006 ▶); Saeedi *et al.* (2010 ▶); Dandia *et al.* (2011 ▶); He *et al.* (2003 ▶).
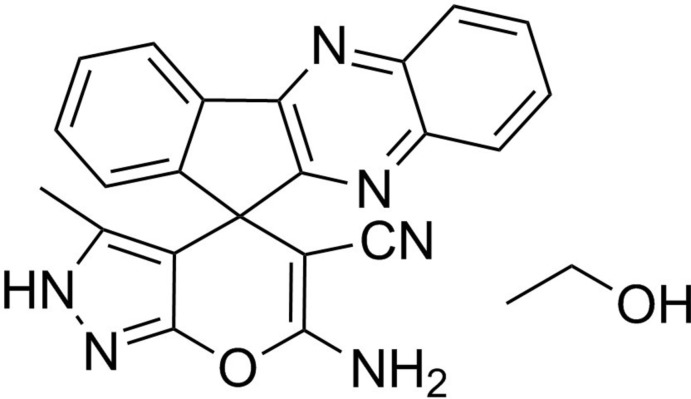


## Experimental

### 

#### Crystal data

C_22_H_14_N_6_O·C_2_H_6_O
*M* *_r_* = 424.46
Monoclinic, 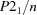

*a* = 14.5060 (6) Å
*b* = 11.1732 (3) Å
*c* = 14.7365 (6) Å
β = 118.859 (5)°
*V* = 2091.84 (13) Å^3^
*Z* = 4
Mo *K*α radiation
μ = 0.09 mm^−1^
*T* = 293 K
0.33 × 0.30 × 0.25 mm

#### Data collection

Agilent Xcalibur Sapphire3 Gemini ultra diffractometer
Absorption correction: multi-scan (*CrysAlis PRO*; Agilent, 2011) ▶ *T*_min_ = 0.994, *T*_max_ = 1.000
8822 measured reflections
4504 independent reflections
3259 reflections with *I* > 2σ(*I*)
*R*_int_ = 0.021

#### Refinement

*R*[*F* ^2^ > 2σ(*F* ^2^)] = 0.047
*wR*(*F* ^2^) = 0.121
*S* = 1.02
4504 reflections
292 parameters
H-atom parameters constrained
Δρ_max_ = 0.23 e Å^−3^
Δρ_min_ = −0.22 e Å^−3^

### 

Data collection: *CrysAlis PRO* (Agilent, 2011) ▶; cell refinement: *CrysAlis PRO*; data reduction: *CrysAlis PRO*; program(s) used to solve structure: *SHELXS97* (Sheldrick, 2008 ▶); program(s) used to refine structure: *SHELXL97* (Sheldrick, 2008 ▶); molecular graphics: *OLEX2* (Dolomanov *et al.*, 2009 ▶); software used to prepare material for publication: *OLEX2*.

## Supplementary Material

Crystal structure: contains datablock(s) I, global. DOI: 10.1107/S1600536812026992/kp2405sup1.cif

Structure factors: contains datablock(s) I. DOI: 10.1107/S1600536812026992/kp2405Isup2.hkl

Additional supplementary materials:  crystallographic information; 3D view; checkCIF report

## Figures and Tables

**Table 1:** Hydrogen-bond geometry (Å, °)

*D*—H⋯*A*	*D*—H	H⋯*A*	*D*⋯*A*	*D*—H⋯*A*
N3—H2*B*⋯N5^i^	0.86	2.22	3.064 (2)	165
N2—H2⋯O2^ii^	0.86	2.00	2.850 (2)	168
O2—H2*C*⋯N6	0.82	2.11	2.917 (2)	166

Symmetry codes: (i) 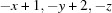; (ii) 
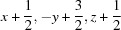
.

**Fig. 1.**
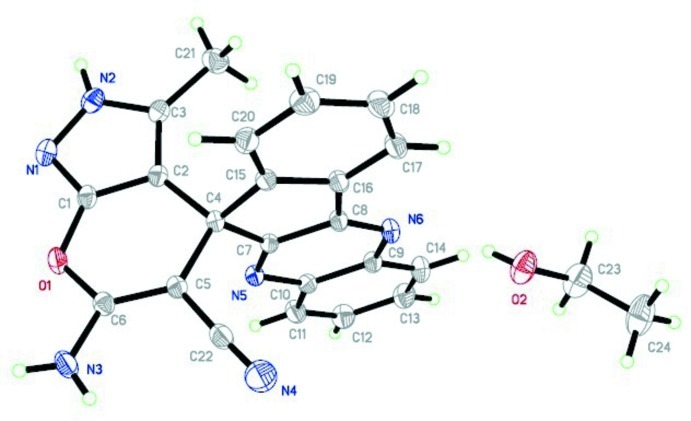
The molecular structure of the title compound in (I) showing the atom numbering scheme. Displacement ellipsoids are drawn at the 50% probability level.

## supplementary crystallographic information

### Comment

Spiro compounds have received considerable interest due to their highly pronounced biological properties (Pradhan *et al.*, 2006); Thus more and more novel spiroheterocycle compounds have been prepared and characterized (Saeedi *et al.*, 2010); Dandia *et al.*, 2011). In addition, quinoxaline derivatives also showed various biological activities (He *et al.*, 2003). Herein, we report the crystal structure of the title compound which is a novel spiroheterocycle containing quinoxaline ring.

The title compound is a formed from the reaciton of 11*H*-indeno [1,2-*b*]quinoxalin-11-one, 3-methyl-2-pyrazolin-5-one and malononitrile. The molecular structure is shown in Fig. 1.

The five member ring (C4, C7, C8, C16, C15) and pyran ring (C4, C5, C6, O1, C1, C2) are perpendicular and the dihedral angle is 89.11 (3)°. N—H···N hydrogen bonding interaction connnects molecules. In addition, solvent molecule (ethanol) is hydrogen bonded by O—H···N and N—H···O (Table 1).

### Experimental

The title compound was prepared by the reaction of 11*H*- indeno[1,2-*b*]quinoxalin-11-one(0.232 g, 1 mmol), 3-methyl-2-pyrazolin- 5-one (0.098 g, 1 mmol) with malononitrile (0.066 g, 1 mmol) in the presence of triethylamine (2 mmol) in ethanol (10.0 ml) under reflux for 1 h. Upon completion (monitored by TLC), the mixture was put into the fridge overnight. Then the reaction mixture was filtered to collect the solid. The crude product was recrystallized from ethanol and then dried to give pure compound (I) in 97% yield (m.p. 532–534 K). Single crystals suitable for X-ray diffraction were obtained by slow evaporation of an ethanol solution of (I) at room temperature.

### Refinement

H atom bound to N was located from a difference Fourier map and refined as riding, with N—H = 0.86 Å, and *U*_iso_(H) = 1.2 *U*_eq_(N), and O(2)—H(2) = 0.82 Å, and *U*_iso_(H) = 1.5 *U*_eq_(O). The remaining H atoms were located in a difference syntheses and refined with C—H = 0.93–0.97 Å and with *U*_iso_(H) = 1.2 *U*_eq_(C).

### Crystal data

**Table:**

C_22_H_14_N_6_O·C_2_H_6_O	*F*(000) = 888
*M**_r_* = 424.46	*D*_x_ = 1.348 Mg m^−^^3^
Monoclinic, *P*2_1_/*n*	Mo *K*α radiation, λ = 0.7107 Å
*a* = 14.5060 (6) Å	Cell parameters from 2373 reflections
*b* = 11.1732 (3) Å	θ = 3.2–29.2°
*c* = 14.7365 (6) Å	µ = 0.09 mm^−^^1^
β = 118.859 (5)°	*T* = 293 K
*V* = 2091.84 (13) Å^3^	Block, colorless
*Z* = 4	0.33 × 0.30 × 0.25 mm

### Data collection

**Table:**

Agilent Xcalibur Sapphire3 Gemini ultra diffractometer	4504 independent reflections
Radiation source: Enhance (Mo) X-ray Source	3259 reflections with *I* > 2σ(*I*)
Graphite monochromator	*R*_int_ = 0.021
Detector resolution: 16.0288 pixels mm^-1^	θ_max_ = 27.0°, θ_min_ = 3.2°
ω scans	*h* = −13→18
Absorption correction: multi-scan (*CrysAlis PRO*; Agilent, 2011)	*k* = −14→12
*T*_min_ = 0.994, *T*_max_ = 1.000	*l* = −18→18
8822 measured reflections	

### Refinement

**Table:**

Refinement on *F*^2^	Primary atom site location: structure-invariant direct methods
Least-squares matrix: full	Secondary atom site location: difference Fourier map
*R*[*F*^2^ > 2σ(*F*^2^)] = 0.047	Hydrogen site location: inferred from neighbouring sites
*wR*(*F*^2^) = 0.121	H-atom parameters constrained
*S* = 1.02	*w* = 1/[σ^2^(*F*_o_^2^) + (0.0506*P*)^2^ + 0.6387*P*] where *P* = (*F*_o_^2^ + 2*F*_c_^2^)/3
4504 reflections	(Δ/σ)_max_ < 0.001
292 parameters	Δρ_max_ = 0.23 e Å^−^^3^
0 restraints	Δρ_min_ = −0.22 e Å^−^^3^

### Special details

**Table:**

Experimental. Absorption correction: CrysAlisPro, Agilent Technologies, Version 1.171.35.15 (release 03-08-2011 CrysAlis171 .NET) (compiled Aug 3 2011,13:03:54) Empirical absorption correction using spherical harmonics, implemented in SCALE3 ABSPACK scaling algorithm.
Geometry. All e.s.d.'s (except the e.s.d. in the dihedral angle between two l.s. planes) are estimated using the full covariance matrix. The cell e.s.d.'s are taken into account individually in the estimation of e.s.d.'s in distances, angles and torsion angles; correlations between e.s.d.'s in cell parameters are only used when they are defined by crystal symmetry. An approximate (isotropic) treatment of cell e.s.d.'s is used for estimating e.s.d.'s involving l.s. planes.
Refinement. Refinement of *F*^2^ against ALL reflections. The weighted *R*-factor *wR* and goodness of fit *S* are based on *F*^2^, conventional *R*-factors *R* are based on *F*, with *F* set to zero for negative *F*^2^. The threshold expression of *F*^2^ > σ(*F*^2^) is used only for calculating *R*-factors(gt) *etc*. and is not relevant to the choice of reflections for refinement. *R*-factors based on *F*^2^ are statistically about twice as large as those based on *F*, and *R*- factors based on ALL data will be even larger.

### Fractional atomic coordinates and isotropic or equivalent isotropic displacement parameters (Å^2^)

**Table:**

	*x*	*y*	*z*	*U*_iso_*/*U*_eq_	
C1	0.49786 (13)	1.04411 (15)	0.17993 (14)	0.0288 (4)	
C2	0.46067 (12)	0.94042 (15)	0.20303 (13)	0.0267 (4)	
C3	0.51266 (13)	0.93684 (15)	0.30993 (13)	0.0308 (4)	
C4	0.38386 (12)	0.85793 (14)	0.12114 (12)	0.0258 (4)	
C5	0.34580 (13)	0.92258 (15)	0.01750 (13)	0.0283 (4)	
C6	0.38757 (13)	1.02560 (15)	0.00320 (13)	0.0292 (4)	
C7	0.43275 (12)	0.73529 (14)	0.12372 (12)	0.0252 (4)	
C8	0.37430 (13)	0.64242 (15)	0.13949 (13)	0.0284 (4)	
C9	0.49107 (13)	0.50506 (15)	0.14294 (13)	0.0306 (4)	
C10	0.54857 (13)	0.59738 (15)	0.12637 (13)	0.0284 (4)	
C11	0.64192 (15)	0.56720 (17)	0.12427 (15)	0.0381 (4)	
H11	0.6802	0.6264	0.1128	0.046*	
C12	0.67633 (15)	0.45132 (17)	0.13902 (16)	0.0419 (5)	
H12	0.7380	0.4324	0.1375	0.050*	
C13	0.61982 (16)	0.36059 (17)	0.15637 (16)	0.0426 (5)	
H13	0.6446	0.2823	0.1670	0.051*	
C14	0.52858 (15)	0.38691 (16)	0.15764 (16)	0.0407 (5)	
H14	0.4910	0.3262	0.1683	0.049*	
C15	0.29206 (12)	0.82089 (15)	0.13901 (12)	0.0279 (4)	
C16	0.28671 (13)	0.69658 (15)	0.14753 (13)	0.0294 (4)	
C17	0.20911 (14)	0.64534 (17)	0.16477 (14)	0.0369 (4)	
H17	0.2051	0.5628	0.1699	0.044*	
C18	0.13808 (14)	0.71996 (19)	0.17413 (15)	0.0420 (5)	
H18	0.0859	0.6872	0.1860	0.050*	
C19	0.14381 (15)	0.84317 (19)	0.16597 (15)	0.0417 (5)	
H19	0.0956	0.8920	0.1728	0.050*	
C20	0.22051 (14)	0.89454 (17)	0.14780 (14)	0.0353 (4)	
H20	0.2236	0.9771	0.1417	0.042*	
C21	0.50729 (17)	0.85130 (19)	0.38457 (15)	0.0466 (5)	
H21A	0.5549	0.8764	0.4541	0.070*	
H21B	0.5266	0.7728	0.3731	0.070*	
H21C	0.4369	0.8495	0.3747	0.070*	
C22	0.26033 (15)	0.87000 (17)	−0.07019 (15)	0.0373 (4)	
C23	0.23737 (17)	0.25182 (19)	0.04394 (19)	0.0577 (6)	
H23A	0.2604	0.2213	0.1132	0.069*	
H23B	0.2944	0.2405	0.0281	0.069*	
C24	0.14610 (19)	0.1839 (2)	−0.0296 (2)	0.0707 (8)	
H24A	0.0898	0.1937	−0.0135	0.106*	
H24B	0.1643	0.1007	−0.0252	0.106*	
H24C	0.1240	0.2125	−0.0985	0.106*	
N4	0.19017 (16)	0.82567 (19)	−0.13887 (14)	0.0647 (6)	
N3	0.35675 (12)	1.08220 (14)	−0.08708 (12)	0.0400 (4)	
H2A	0.3060	1.0537	−0.1432	0.048*	
H2B	0.3877	1.1471	−0.0889	0.048*	
N5	0.51689 (10)	0.71530 (12)	0.11524 (11)	0.0282 (3)	
N6	0.39997 (11)	0.52895 (12)	0.14725 (12)	0.0329 (3)	
N1	0.56749 (11)	1.10402 (13)	0.26111 (12)	0.0364 (4)	
N2	0.57499 (11)	1.03496 (13)	0.34080 (12)	0.0366 (4)	
H2	0.6157	1.0523	0.4049	0.044*	
O1	0.46733 (9)	1.08757 (10)	0.08218 (10)	0.0345 (3)	
O2	0.21583 (11)	0.37477 (12)	0.04178 (12)	0.0505 (4)	
H2C	0.2702	0.4107	0.0801	0.076*	

### Atomic displacement parameters (Å^2^)

**Table:**

	*U*^11^	*U*^22^	*U*^33^	*U*^12^	*U*^13^	*U*^23^
C1	0.0296 (8)	0.0220 (8)	0.0361 (9)	0.0013 (7)	0.0169 (7)	0.0006 (8)
C2	0.0269 (8)	0.0229 (8)	0.0309 (9)	−0.0005 (7)	0.0144 (7)	−0.0010 (7)
C3	0.0301 (8)	0.0280 (9)	0.0328 (9)	0.0008 (7)	0.0140 (7)	−0.0021 (8)
C4	0.0288 (8)	0.0219 (8)	0.0276 (8)	−0.0022 (7)	0.0144 (7)	0.0013 (7)
C5	0.0310 (8)	0.0247 (9)	0.0289 (9)	0.0000 (7)	0.0142 (7)	0.0022 (7)
C6	0.0317 (8)	0.0251 (9)	0.0342 (9)	0.0040 (7)	0.0186 (8)	0.0034 (8)
C7	0.0284 (8)	0.0227 (8)	0.0217 (8)	−0.0037 (7)	0.0099 (6)	0.0002 (7)
C8	0.0319 (8)	0.0241 (9)	0.0281 (9)	−0.0039 (7)	0.0137 (7)	0.0020 (7)
C9	0.0345 (9)	0.0253 (9)	0.0304 (9)	−0.0017 (7)	0.0144 (7)	0.0010 (8)
C10	0.0329 (9)	0.0252 (9)	0.0257 (8)	−0.0002 (7)	0.0130 (7)	0.0014 (7)
C11	0.0407 (10)	0.0347 (10)	0.0444 (11)	−0.0007 (8)	0.0250 (9)	0.0022 (9)
C12	0.0408 (10)	0.0381 (11)	0.0506 (12)	0.0051 (9)	0.0251 (9)	−0.0002 (10)
C13	0.0496 (11)	0.0282 (10)	0.0486 (12)	0.0086 (9)	0.0226 (10)	0.0032 (9)
C14	0.0453 (11)	0.0258 (9)	0.0511 (12)	−0.0014 (8)	0.0233 (10)	0.0036 (9)
C15	0.0288 (8)	0.0291 (9)	0.0238 (8)	−0.0044 (7)	0.0111 (7)	−0.0004 (7)
C16	0.0314 (8)	0.0282 (9)	0.0279 (9)	−0.0047 (7)	0.0136 (7)	0.0017 (8)
C17	0.0372 (10)	0.0372 (10)	0.0380 (10)	−0.0079 (8)	0.0194 (8)	0.0023 (9)
C18	0.0360 (10)	0.0516 (12)	0.0444 (11)	−0.0086 (9)	0.0241 (9)	−0.0007 (10)
C19	0.0363 (10)	0.0488 (12)	0.0458 (11)	0.0013 (9)	0.0244 (9)	−0.0040 (10)
C20	0.0373 (10)	0.0315 (10)	0.0381 (10)	−0.0010 (8)	0.0189 (8)	−0.0017 (8)
C21	0.0502 (12)	0.0511 (13)	0.0346 (10)	−0.0036 (10)	0.0173 (9)	0.0055 (10)
C22	0.0422 (10)	0.0358 (10)	0.0322 (10)	−0.0041 (9)	0.0165 (8)	0.0074 (9)
C23	0.0507 (13)	0.0424 (13)	0.0596 (14)	0.0070 (10)	0.0105 (11)	0.0025 (11)
C24	0.0598 (14)	0.0394 (13)	0.0858 (19)	0.0004 (11)	0.0136 (13)	−0.0012 (13)
N4	0.0644 (12)	0.0708 (14)	0.0390 (10)	−0.0235 (11)	0.0091 (9)	0.0001 (10)
N3	0.0466 (9)	0.0348 (9)	0.0392 (9)	−0.0034 (7)	0.0211 (8)	0.0106 (8)
N5	0.0321 (7)	0.0245 (7)	0.0282 (7)	−0.0022 (6)	0.0147 (6)	0.0014 (6)
N6	0.0364 (8)	0.0239 (8)	0.0394 (9)	−0.0036 (6)	0.0190 (7)	0.0028 (7)
N1	0.0347 (8)	0.0282 (8)	0.0432 (9)	−0.0042 (7)	0.0163 (7)	−0.0042 (7)
N2	0.0335 (8)	0.0345 (9)	0.0347 (8)	−0.0033 (7)	0.0108 (7)	−0.0064 (7)
O1	0.0386 (7)	0.0250 (6)	0.0405 (7)	−0.0053 (5)	0.0196 (6)	0.0037 (6)
O2	0.0425 (8)	0.0366 (8)	0.0520 (9)	−0.0002 (6)	0.0065 (7)	−0.0011 (7)

### Geometric parameters (Å, º)

**Table:**

C1—C2	1.388 (2)	C13—C14	1.365 (3)
C1—N1	1.316 (2)	C14—H14	0.9300
C1—O1	1.375 (2)	C15—C16	1.400 (2)
C2—C3	1.380 (2)	C15—C20	1.379 (2)
C2—C4	1.500 (2)	C16—C17	1.391 (2)
C3—C21	1.487 (3)	C17—H17	0.9300
C3—N2	1.352 (2)	C17—C18	1.383 (3)
C4—C5	1.531 (2)	C18—H18	0.9300
C4—C7	1.535 (2)	C18—C19	1.388 (3)
C4—C15	1.534 (2)	C19—H19	0.9300
C5—C6	1.364 (2)	C19—C20	1.388 (3)
C5—C22	1.416 (2)	C20—H20	0.9300
C6—N3	1.340 (2)	C21—H21A	0.9600
C6—O1	1.368 (2)	C21—H21B	0.9600
C7—C8	1.428 (2)	C21—H21C	0.9600
C7—N5	1.305 (2)	C22—N4	1.145 (2)
C8—C16	1.463 (2)	C23—H23A	0.9700
C8—N6	1.311 (2)	C23—H23B	0.9700
C9—C10	1.420 (2)	C23—C24	1.455 (3)
C9—C14	1.404 (2)	C23—O2	1.406 (2)
C9—N6	1.379 (2)	C24—H24A	0.9600
C10—C11	1.410 (2)	C24—H24B	0.9600
C10—N5	1.379 (2)	C24—H24C	0.9600
C11—H11	0.9300	N3—H2A	0.8600
C11—C12	1.367 (3)	N3—H2B	0.8600
C12—H12	0.9300	N1—N2	1.365 (2)
C12—C13	1.403 (3)	N2—H2	0.8600
C13—H13	0.9300	O2—H2C	0.8200
			
N1—C1—C2	114.84 (16)	C20—C15—C16	120.46 (16)
N1—C1—O1	119.36 (15)	C15—C16—C8	108.37 (14)
O1—C1—C2	125.79 (15)	C17—C16—C8	130.96 (16)
C1—C2—C4	122.78 (15)	C17—C16—C15	120.62 (16)
C3—C2—C1	103.96 (15)	C16—C17—H17	120.7
C3—C2—C4	133.23 (15)	C18—C17—C16	118.53 (18)
C2—C3—C21	131.90 (17)	C18—C17—H17	120.7
N2—C3—C2	105.59 (15)	C17—C18—H18	119.7
N2—C3—C21	122.50 (16)	C17—C18—C19	120.70 (17)
C2—C4—C5	106.43 (13)	C19—C18—H18	119.7
C2—C4—C7	111.95 (13)	C18—C19—H19	119.5
C2—C4—C15	113.39 (13)	C18—C19—C20	120.93 (18)
C5—C4—C7	112.69 (13)	C20—C19—H19	119.5
C5—C4—C15	111.93 (13)	C15—C20—C19	118.76 (17)
C15—C4—C7	100.60 (12)	C15—C20—H20	120.6
C6—C5—C4	125.38 (15)	C19—C20—H20	120.6
C6—C5—C22	117.88 (15)	C3—C21—H21A	109.5
C22—C5—C4	116.73 (14)	C3—C21—H21B	109.5
C5—C6—O1	123.50 (15)	C3—C21—H21C	109.5
N3—C6—C5	126.27 (16)	H21A—C21—H21B	109.5
N3—C6—O1	110.22 (14)	H21A—C21—H21C	109.5
C8—C7—C4	110.44 (14)	H21B—C21—H21C	109.5
N5—C7—C4	126.36 (14)	N4—C22—C5	177.6 (2)
N5—C7—C8	123.18 (15)	H23A—C23—H23B	107.9
C7—C8—C16	108.64 (14)	C24—C23—H23A	109.1
N6—C8—C7	123.45 (15)	C24—C23—H23B	109.1
N6—C8—C16	127.90 (15)	O2—C23—H23A	109.1
C14—C9—C10	119.56 (16)	O2—C23—H23B	109.1
N6—C9—C10	121.52 (15)	O2—C23—C24	112.35 (18)
N6—C9—C14	118.91 (15)	C23—C24—H24A	109.5
C11—C10—C9	118.69 (16)	C23—C24—H24B	109.5
N5—C10—C9	121.65 (15)	C23—C24—H24C	109.5
N5—C10—C11	119.63 (15)	H24A—C24—H24B	109.5
C10—C11—H11	119.9	H24A—C24—H24C	109.5
C12—C11—C10	120.26 (17)	H24B—C24—H24C	109.5
C12—C11—H11	119.9	C6—N3—H2A	120.0
C11—C12—H12	119.6	C6—N3—H2B	120.0
C11—C12—C13	120.85 (18)	H2A—N3—H2B	120.0
C13—C12—H12	119.6	C7—N5—C10	115.20 (14)
C12—C13—H13	119.9	C8—N6—C9	114.91 (14)
C14—C13—C12	120.18 (17)	C1—N1—N2	101.65 (14)
C14—C13—H13	119.9	C3—N2—N1	113.96 (14)
C9—C14—H14	119.8	C3—N2—H2	123.0
C13—C14—C9	120.44 (17)	N1—N2—H2	123.0
C13—C14—H14	119.8	C6—O1—C1	115.14 (13)
C16—C15—C4	111.92 (14)	C23—O2—H2C	109.5
C20—C15—C4	127.62 (15)		
			
C1—C2—C3—C21	178.33 (19)	C8—C16—C17—C18	176.67 (17)
C1—C2—C3—N2	−0.36 (18)	C9—C10—C11—C12	−0.6 (3)
C1—C2—C4—C5	−9.4 (2)	C9—C10—N5—C7	1.7 (2)
C1—C2—C4—C7	114.17 (17)	C10—C9—C14—C13	0.2 (3)
C1—C2—C4—C15	−132.85 (16)	C10—C9—N6—C8	−2.8 (2)
C1—N1—N2—C3	0.01 (19)	C10—C11—C12—C13	0.0 (3)
C2—C1—N1—N2	−0.26 (19)	C11—C10—N5—C7	−176.50 (16)
C2—C1—O1—C6	5.1 (2)	C11—C12—C13—C14	0.7 (3)
C2—C3—N2—N1	0.24 (19)	C12—C13—C14—C9	−0.8 (3)
C2—C4—C5—C6	9.6 (2)	C14—C9—C10—C11	0.5 (3)
C2—C4—C5—C22	−170.60 (15)	C14—C9—C10—N5	−177.66 (16)
C2—C4—C7—C8	121.49 (15)	C14—C9—N6—C8	175.74 (17)
C2—C4—C7—N5	−57.1 (2)	C15—C4—C5—C6	134.00 (17)
C2—C4—C15—C16	−121.20 (16)	C15—C4—C5—C22	−46.2 (2)
C2—C4—C15—C20	58.1 (2)	C15—C4—C7—C8	0.76 (16)
C3—C2—C4—C5	172.97 (17)	C15—C4—C7—N5	−177.86 (15)
C3—C2—C4—C7	−63.5 (2)	C15—C16—C17—C18	−0.5 (3)
C3—C2—C4—C15	49.5 (2)	C16—C8—N6—C9	−175.98 (16)
C4—C2—C3—C21	−3.7 (3)	C16—C15—C20—C19	0.5 (3)
C4—C2—C3—N2	177.61 (17)	C16—C17—C18—C19	0.3 (3)
C4—C5—C6—N3	178.60 (16)	C17—C18—C19—C20	0.3 (3)
C4—C5—C6—O1	−2.9 (3)	C18—C19—C20—C15	−0.7 (3)
C4—C5—C22—N4	25 (6)	C20—C15—C16—C8	−177.64 (15)
C4—C7—C8—C16	0.18 (18)	C20—C15—C16—C17	0.1 (3)
C4—C7—C8—N6	−178.41 (16)	C21—C3—N2—N1	−178.61 (16)
C4—C7—N5—C10	176.18 (15)	C22—C5—C6—N3	−1.2 (3)
C4—C15—C16—C8	1.7 (2)	C22—C5—C6—O1	177.29 (16)
C4—C15—C16—C17	179.49 (15)	N3—C6—O1—C1	173.63 (14)
C4—C15—C20—C19	−178.78 (16)	N5—C7—C8—C16	178.85 (15)
C5—C4—C7—C8	−118.58 (15)	N5—C7—C8—N6	0.3 (3)
C5—C4—C7—N5	62.8 (2)	N5—C10—C11—C12	177.58 (17)
C5—C4—C15—C16	118.38 (16)	N6—C8—C16—C15	177.34 (17)
C5—C4—C15—C20	−62.3 (2)	N6—C8—C16—C17	−0.1 (3)
C5—C6—O1—C1	−5.1 (2)	N6—C9—C10—C11	179.10 (16)
C6—C5—C22—N4	−155 (6)	N6—C9—C10—N5	0.9 (3)
C7—C4—C5—C6	−113.47 (18)	N6—C9—C14—C13	−178.41 (18)
C7—C4—C5—C22	66.33 (19)	N1—C1—C2—C3	0.4 (2)
C7—C4—C15—C16	−1.51 (18)	N1—C1—C2—C4	−177.84 (15)
C7—C4—C15—C20	177.78 (16)	N1—C1—O1—C6	−174.05 (15)
C7—C8—C16—C15	−1.16 (19)	O1—C1—C2—C3	−178.79 (15)
C7—C8—C16—C17	−178.62 (17)	O1—C1—C2—C4	3.0 (3)
C7—C8—N6—C9	2.3 (2)	O1—C1—N1—N2	179.00 (14)
C8—C7—N5—C10	−2.3 (2)		

### Hydrogen-bond geometry (Å, º)

**Table:**

*D*—H···*A*	*D*—H	H···*A*	*D*···*A*	*D*—H···*A*
N3—H2*B*···N5^i^	0.86	2.22	3.064 (2)	165
N2—H2···O2^ii^	0.86	2.00	2.850 (2)	168
O2—H2*C*···N6	0.82	2.11	2.917 (2)	166

Symmetry codes: (i) −*x*+1, −*y*+2, −*z*; (ii) *x*+1/2, −*y*+3/2, *z*+1/2.
